# Nesfatin-1 protects dopaminergic neurons against MPP^+^/MPTP-induced neurotoxicity through the C-Raf–ERK1/2-dependent anti-apoptotic pathway

**DOI:** 10.1038/srep40961

**Published:** 2017-01-20

**Authors:** Xiao-Li Shen, Ning Song, Xi-Xun Du, Yong Li, Jun-Xia Xie, Hong Jiang

**Affiliations:** 1 Department of Physiology, Shandong Provincial Key Laboratory of Pathogenesis and Prevention of Neurological Disorders and State Key Disciplines: Physiology, Medical College of Qingdao University, Qingdao, 266071, China

## Abstract

Several brain-gut peptides have been reported to have a close relationship with the central dopaminergic system; one such brain-gut peptide is nesfatin-1. Nesfatin-1 is a satiety peptide that is predominantly secreted by X/A-like endocrine cells in the gastric glands, where ghrelin is also secreted. We previously reported that ghrelin exerted neuroprotective effects on nigral dopaminergic neurons, which implied a role for ghrelin in Parkinson’s disease (PD). In the present study, we aim to clarify whether nesfatin-1 has similar effects on dopaminergic neurons both *in vivo* and *in vitro.* We show that nesfatin-1 attenuates the loss of nigral dopaminergic neurons in the 1-methyl-4-phenyl-1, 2,3,6-tetrahydropyridine (MPTP)-induced mouse model of PD. In addition, nesfatin-1 antagonized 1-methyl-4-phenylpyridillium ion (MPP^+^)-induced toxicity by restoring mitochondrial function, inhibiting cytochrome C release and preventing caspase-3 activation in MPP^+^-treated MES23.5 dopaminergic cells. These neuroprotective effects could be abolished by selective inhibition of C-Raf and the extracellular signal-regulated protein kinase 1/2 (ERK1/2). Our data suggest that C-Raf-ERK1/2, which is involved in an anti-apoptotic pathway, is responsible for the neuroprotective effects of nesfatin-1 in the context of MPTP-induced toxicity. These results imply that nesfatin-1 might have therapeutic potential for PD.

Recently, several brain-gut peptides, such as neurotensin, ghrelin, and glucagon-like peptide-1, have been reported to have a close relationship with the central dopaminergic system^1^,^2^. The degeneration of nigral dopaminergic neurons is associated with Parkinson’s disease (PD), which is characterized by stiffness, tremors, slowness of movement, and postural instability^3^,^4^,^5^. Thus far, there have been no satisfactory strategies that slow down the neurodegeneration of dopaminergic neurons in PD. Many studies have demonstrated that apoptosis induced by mitochondrial dysfunction plays important roles in the development of PD^6^,^7^,^8^, indicating that the prevention of apoptosis could be a therapeutic strategy for PD. In our previous studies, we reported that ghrelin, which is secreted by X/A-like cells in the gastric glands, showed neuroprotective effects on dopaminergic neurons exposed to the toxin 1-methyl-4-phenyl-1, 2,3,6-tetrahydropyridine (MPTP) both *in vivo* and *in vitro* via an anti-apoptotic effect mediated by the ghrelin-protein kinase C (PKC) signaling pathway^9^,^10^,^11^.

Nesfatin-1 co-localizes with ghrelin and is also secreted by X/A-like endocrine cells^12^. It is an 82-amino acids protein that was discovered in 2006. Nesfatin-1 is encoded by the NEFA gene (also known as NUCB2) and has been shown to have anorexigenic properties^13^,^14^,^15^. It was also found to cross the blood-brain barrier in a bidirectional manner^16^. Nesfatin-1 has been identified not only in peripheral tissues, including the gastric glands, duodenum, and pancreatic islets, but also in the central nervous system, including cortical areas, the limbic system, the thalamus, and the hypothalamus^17^,^18^,^19^. In addition to playing opposing roles to ghrelin in the hypothalamic control of food intake, body-weight, and energy homeostasis, nesfatin-1 was also reported to be involved in reproduction, sleep, anxiety, and stress-related responses^20^,^21^,^22^,^23^,^24^. Unlike the stimulation of appetite and food intake induced by ghrelin, nesfatin-1 inhibited food intake and decreased weight gain in rats^13^,^15^,^25^. However, peripheral use of nesfatin-1 showed anti-apoptotic and anti-inflammatory effects similar to ghrelin in the settings of subarachnoid hemorrhage and brain injury^26^,^27^,^28^,^29^. We have recently reported that nesfatin-1 can antagonize rotenone-induced neurotoxicity in MES23.5 dopaminergic cells via anti-apoptotic effects^30^. Although our data, together with data from other groups, showed that nesfatin-1 could exert various effects in the central nervous system, its receptors have yet to be cloned, although they are speculated to be G-protein-coupled receptors acting through Gi and Gs^17^,^31^. This lack of knowledge about nesfatin-1 receptors makes it quite difficult to decipher the central role of nesfatin-1. Fortunately, our previous study using the patch clamp technique found that nesfatin-1 could directly decrease the excitability of nigral dopaminergic neurons in rat brain slices^32^, suggesting that the nesfatin-1 receptor might be expressed by nigral dopaminergic neurons.

In the present study, we sought to study the effect of nesfatin-1 on nigral dopaminergic neurons in PD. The MPTP-induced mouse model of PD and the 1-methyl-4-phenylpyridillium ion (MPP^+^)-induced cell model of PD were employed for *in vivo* and *in vitro* studies, respectively. The possible underlying mechanisms were also investigated.

## Results

### Nesfatin-1 attenuated MPTP-induced depletion of dopamine and its metabolites in the striatum of mice

**Figure 1:**
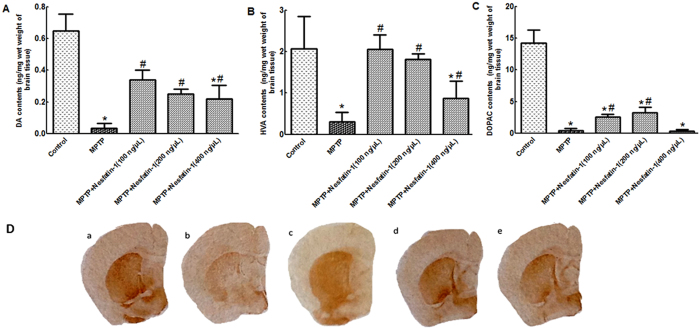
Nesfatin-1 attenuated MPTP-induced depletion of DA and its metabolites in the striatum (n = 6). (**A**) Striatal DA levels in control mice, mice treated with MPTP alone, and mice treated with MPTP+nesfatin-1 (100 ng, 200 ng, and 400 ng/mouse). (**B**) Striatal HVA levels in the different groups. (**C**) Striatal DOPAC levels in the different groups. Each value represents the mean ± SEM. (**D**) The effects of nesfatin-1 on the number of TH-immunoreactive fibers in the striatum. (a) Control treatment, (b) MPTP alone, (c) MPTP+nesfatin-1 (100 ng/mouse), (d) MPTP+nesfatin-1 (200 ng/mouse), and (e) MPTP+nesfatin-1 (400 ng/mouse). ^*^P < 0.01, compared with the control group. ^#^*P* < 0.05, compared with the MPTP group. (DOPAC: dihydroxyphenylacetic acid, HVA: homovanillic acid, MPTP: 1-methyl-4-phenyl-1,2,3,6-tetrahydropyridine, Str: Striatum, TH: tyrosine hydroxylase).

After 5 days of MPTP injections, levels of dopamine (DA) and its metabolites dihydroxyphenylacetic acid (DOPAC) and homovanillic acid (HVA) were decreased in the striatum in the MPTP treatment group. Nesfatin-1 conferred a significant protective effect against MPTP-induced DA depletion. Compared with control treatment, MPTP treatment induced a 96.61% depletion of DA. The DA levels in mice pretreated with 100 ng, 200 ng, and 400 ng nesfatin-1 were restored to 52%, 39%, and 34%, respectively, of the DA levels in the control group. Both 100 ng and 200 ng nesfatin-1 also restored striatal DOPAC and HVA levels, whereas 400 ng nesfatin-1 only increased the levels of HVA (Fig. 1A,B and C). In accordance with the above results, immunohistochemistry staining of the stiatum showed that the number of tyrosine hydroxylase (TH)-immunoreactivity (TH+) fibers was markedly reduced in mice after 5 days of MPTP injections; pretreatment of mice with 100 ng, 200 ng, and 400 ng nesfatin-1protected most TH+fibers from MPTP-induced toxicity (Fig. 1D).

### Nesfatin-1 protected nigral dopaminergic neurons against MPTP-induced neurotoxicity in mice

**Figure 2:**
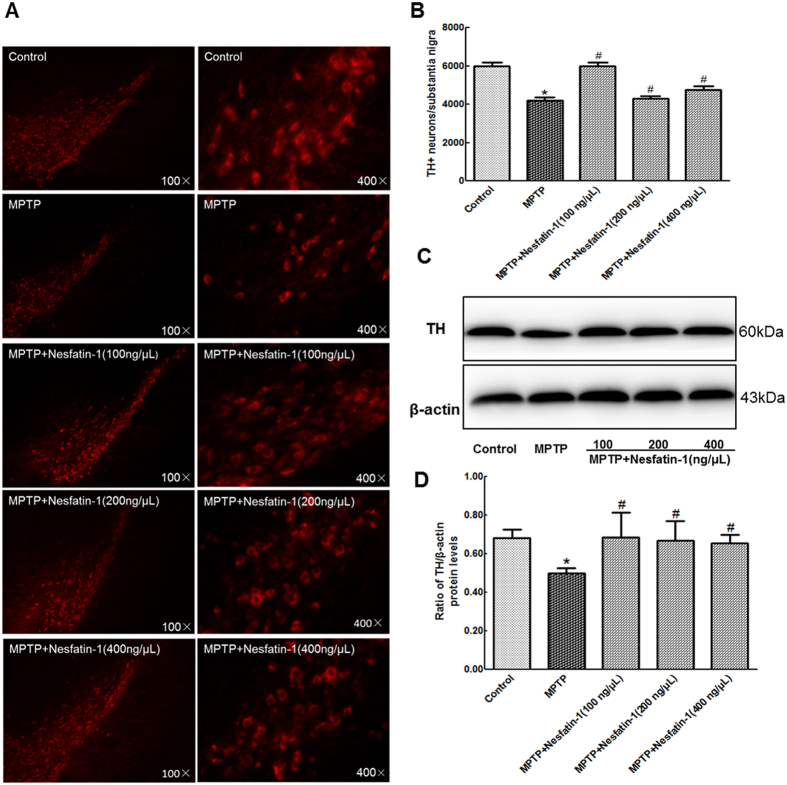
Nesfatin-1 protected nigral dopaminergic neurons against MPTP-induced neurotoxicity (n = 6). (**A**) TH+neurons in different groups were shown. (**B**) A summary of the data showed the numbers of TH+neurons in different groups. (**C**) An original figure of a western blot showing TH protein levels in different groups. (**D**) Statistical analysis of TH protein levels in different groups. Each value represents the mean ± SEM. ^*^P < 0.05, compared with the control group. ^#^*P* < 0.05, compared with the MPTP group. Full-length blots are presented in Supplementary Figure S2. (MPTP: 1-methyl-4-phenyl-1,2,3,6-tetrahydropyridine, TH:tyrosine hydroxylase).

After 5 days of MPTP treatment, mice showed a significant loss of TH + neurons and decreased TH protein levels in the substantia nigra. Compared with that of the control, the survival ratio of TH + neurons in the substantia nigra decreased to 70% and TH protein levels decreased to 63% in the MPTP-treated group. Pretreatment with nesfatin-1 had a significant protective effect on dopaminergic neurons in mice receiving MPTP treatment. It was observed that 100 ng, 200 ng, and 400 ng nesfatin-1 preserved as many as 100%, 82%, and 80% of TH + neurons (Fig. 2A and B), and 100%, 98%, and 95% of TH protein levels in the substantia nigra (Fig. 2C and D, also Supplementary Figure S2), respectively, compared with controls.

### Nesfatin-1 had no effect on MPTP metabolism in the striatum

**Figure 3:**
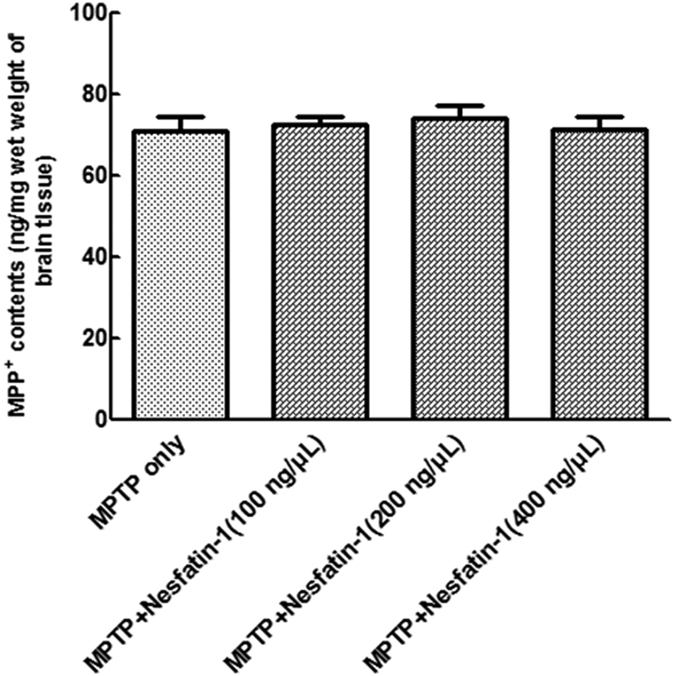
Nesfatin-1 had no effect on MPTP metabolism in striatum. For the detection of MPP^+^ levels in striatum, mice were treated with vehicle or nesfatin-1 (ICV) 20 min before MPTP (i.p.) injection. MPP^**+**^ levels were detected 90 min after MPTP injection. Each value represents the mean ± SEM. All presented values do not significantly differ from MPTP group. (ICV: intracerebroyentricular injection, i.p.: intraperitoneal injection, MPTP: 1-methyl-4-phenyl-1, 2, 3, 6-tetrahydropyridine).

MPTP is catalyzed to MPP^+^ by monoamine oxidase B in neurogliocyte^33^. In order to illustrate that the neuroprotective effects of nesfatin-1 were not caused by its effect on MPTP metabolism, we measured the striatal levels of MPP^+^ 90 min after MPTP injection. As shown in Fig. 3, nesfatin-1 pretreatment 20 min had no effect on the levels of striatal MPP^+^ in MPTP-treated mice. These data suggest that the neuroprotective effect of nesfatin-1 was not caused by its effect on the conversion of MPTP to MPP^+^.

### Nesfatin-1 antagonized MPP^+^-induced cytotoxicity in MES23.5 cells

**Figure 4:**
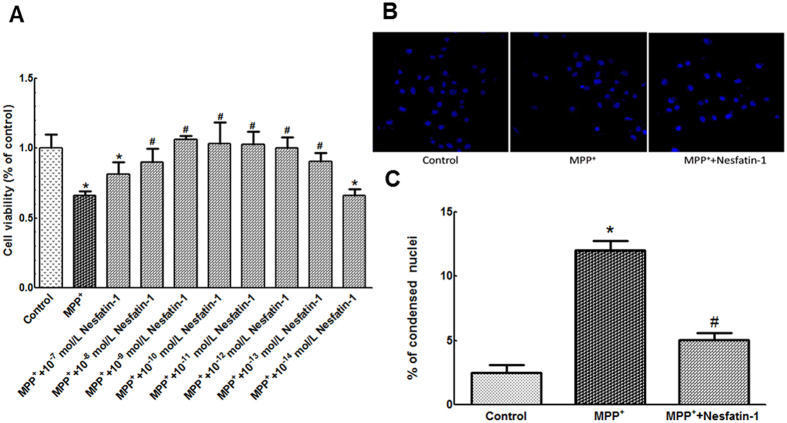
Nesfatin-1 antagonized MPP^+^-induced reduction in cell viability. (**A**) Pre-incubation with nesfatin-1 (10^−13^ to 10^−8^ mol/L) antagonized MPP^**+**^-induced reduction in cell viability, as determined using MTT assay. (**B**,**C**) Pretreatment with nesfatin-1(10^−9^ mol/L) for 20 min significantly attenuated MPP^**+**^-induced nuclear hypercondensation, breakage, and anachromasis. Magnification × 100 and × 400. Data are presented as the mean ± SEM of six independent experiments. ^*^P < 0.05 compared with the control. ^#^*P* < 0.05 compared with the MPP^**+**^-treated group. (MPP^**+**^:1-methyl-4-phenylpyridillium ion, MTT:Methyl thiazolyltetrazolium).

A significant reduction in cell viability was observed after treatment with 300 μmol/L MPP^+^, whereas pretreatment with nesfatin-1 (10^−13^–10^−8^ mol/L) significantly restored cell viability. Compared with 66.21% viability (normalized to the control group) in the MPP^+^-treated group, the viability was restored to 90.81%, 100.11%, 102.80%, 103.11%, 106.29%, and 90.19% in the 10^−13^, 10^−12^, 10^−11^, 10^−10^, 10^−9^, and 10^−8^ mol/L nesfatin-1-pretreated groups, respectively. 10^−9^ mol/L nesfatin-1 showed the best efficacy, while 10^−7^ and 10^−14^ mol/L nesfatin-1 were not efficacious (Fig. 4A). Treatment with MPP^+^ (300 μmol/L) also induced hypercondensation, breakage, and anachromasis of nuclei in MES23.5 cells, whereas pretreatment with 10^−9^ mol/L nesfatin-1 for 20 min attenuated the morphological changes induced by MPP^+^ in these cells (Fig. 4B and C).

### Nesfatin-1 antagonized MPP^+^-induced cytotoxicity in MES23.5 cells by having an anti-apoptotic action

**Figure 5:**
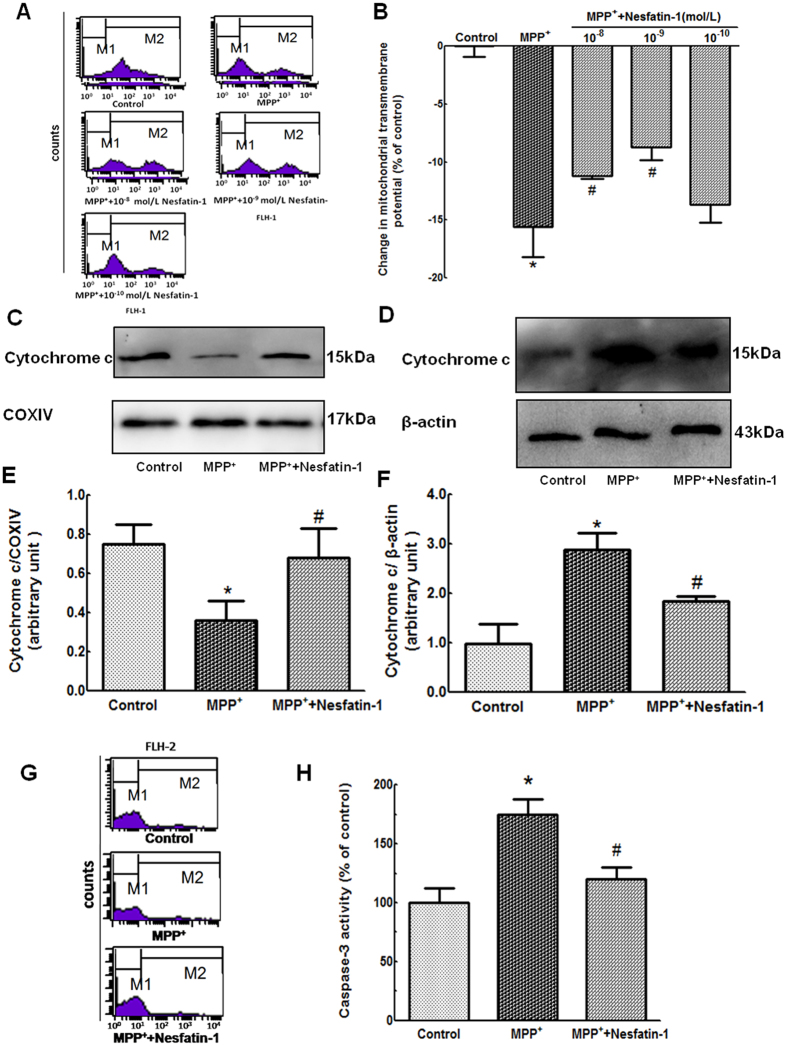
Nesfatin-1 antagonized MPP^+^-induced apoptosis by attenuating mitochondrial dysfunction in MES23.5 cells. (**A**,**B**) Pretreatment with nesfatin-1 (10^−9^ and 10^−8^ mol/L) for 20 min antagonized MPP^**+**^-induced collapse of the ΔΨm, as detected by flow cytometry. Data are presented as the percentage of control (which was set at 100%). (**C**,**D**,**E**,**F**) Pretreatment with nesfatin-1 (10^−9^ mol/L) prevented MPP^**+**^-induced cytochrome C release from the mitochondria into the cytoplasm. (**G**,**H**) MPP^**+**^-induced caspase-3 activation was abolished by pretreatment with nesfatin-1 (10^−9^ mol/L) for 20 min. Data are presented as the percentage of control (which was set at 100%). Data are presented as the mean ± SEM of six independent experiments. ^*^P < 0.05 compared with the control. ^#^*P* < 0.05 compared with the MPP^**+**^-treated group. Full-length blots are presented in Supplementary Figure S5. (MPP^**+**^:1-methyl-4-phenylpyridillium ion, ΔΨm: mitochondrial transmembrane potential).

Changes in mitochondrial transmembrane potential (ΔΨm) are a marker of mitochondrial function, which is involved in apoptosis. After pretreatment with different concentrations of nesfatin-1 (10^−8^ to 10^−10^ mol/L) for 20 min, MES23.5 cells were treated with MPP^+^ at a final concentration 300 μmol/L in serum-starved medium for an additional 24 hrs. As shown in Fig. 5A and B, an apparent collapse in ΔΨm was observed in MPP^+^-treated cells, while a recovery of ΔΨM was observed in cells pretreated with nesfatin-1 (10^−9^ and 10^−8^ mol/L) for 20 min. Nesfatin-1 at 10^−9^ mol/L exerted the highest efficacy. These results indicated that nesfatin-1 antagonized the MPP^+^-induced ΔΨm collapse in MES23.5 cells.

Mitochondrial dysfunction often leads to cytochrome C leakage from the mitochondria into the cytoplasm. The mitochondrial cytochrome C protein level was decreased to 48% in cells treated with 300 μmol/L MPP^+^, while pretreatment with 10^−9^ mol/L nesfatin-1 upregulated the protein level of mitochondrial cytochrome C to 91% of the level found in the controls (Fig. 5C and E). Furthermore, compared with that of the control, MPP^+^ treatment caused an almost three-fold increased expression of cytochrome C in the cytoplasm. The cytoplasmic cytochrome C levels were significantly decreased after pretreatment with 10^−9^ mol/L nesfatin-1 (Fig. 5D and F, also Supplementary Figure S5). These results suggested that nesfatin-1 attenuated MPP^+^ -induced cytochrome C release from the mitochondria into the cytoplasm in MES23.5 cells.

Caspase-3 activation is a key marker of cell apoptosis. After pretreatment with nesfatin-1 (10^−9^ mol/L) for 20 min, cells were incubated with MPP^+^ (300 μmol/L) for 24 hrs. As shown in Fig. 5G and H, MPP^+^ treatment induced significant activation of caspase-3 in MES23.5 cells, and this was attenuated by pretreatment with 10^−9^ mol/L nesfatin-1.

### C-Raf and ERK1/2 were involved in the anti-apoptotic action of nesfatin-1

**Figure 6:**
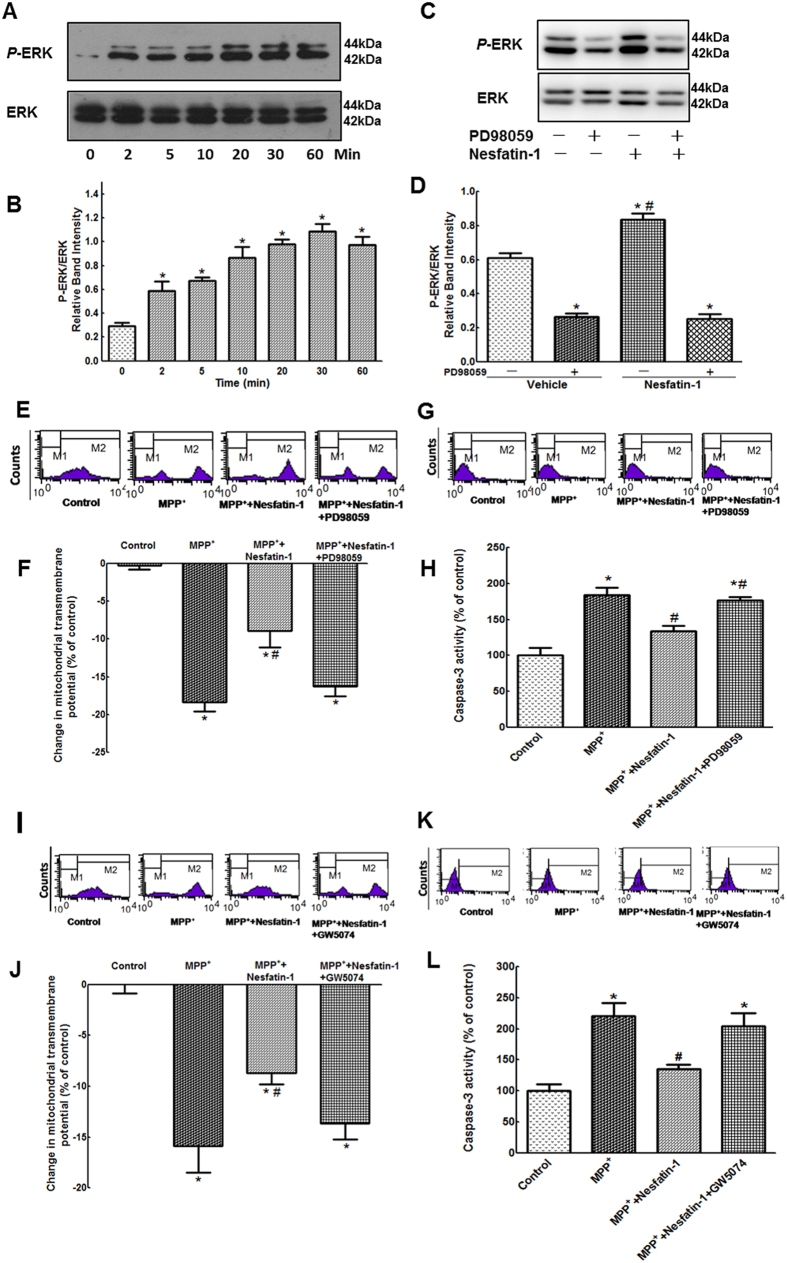
The C-Raf-ERK1/2 pathway was involved in the anti-apoptotic action of nesfatin-1 in MES23.5 cells. (**A**,**B**) Nesfatin-1 (10^−9^ mol/L) stimulated phosphorylation of ERK1/2 after 2, 5, 10, 20, 30, and 60 min, with levels of phosphorylation peaking at 30 min. (**C**,**D**) Pretreatment with the ERK1/2 inhibitor PD98059 (10 μmol/L) abolished nesfatin-1-induced ERK1/2 phosphorylation. (**E**,**F**) Pretreatment with PD98059 (10 μmol/L) abolished the protective effect of nesfatin-1 (10^−9^ mol/L) on the collapse of the ΔΨm induced by MPP^**+**^. (**G**,**H**) Pretreatment with PD98059 (10 μmol/L) abolished the protective effect of nesfatin-1 (10^−9^ mol/L) on caspase-3 activation induced by MPP^**+**^. (**I**,**J**) Pretreatment with the C-Raf inhibitor GW5074 (5 μmol/L) abolished the protective efficacy of nesfatin-1 (10^−9^ mol/L) on the MPP^**+**^-induced collapse of the ΔΨm. (**K**,**L**) Pretreatment with GW5074 (5 μmol/L) abolished the protective effect of nesfatin-1 (10^−9^ mol/L) on caspase-3 activation induced by MPP^**+**^. ^*^P < 0.05 compared with the control group. ^#^*P* < 0.05 compared with the MPP^**+**^-treated group. Full-length blots are presented in Supplementary Figure S6. (MPP^**+**^:1-methyl-4-phenylpyridillium ion, ERK1/2: extracellular signal-regulated protein kinase 1/2, ΔΨm: mitochondrial transmembrane potential).

Extracellular signal-regulated kinases (ERKs) are known to be linked to cell survival and have neuroprotective effects^34^. To elucidate whether the anti-apoptotic effects of nesfatin-1 were due to ERK activation, we evaluated the effect of nesfatin-1 on the activation of ERK1/2 in MES23.5 cells. As shown in Fig. 6A and B (also Supplementary Figure S6), phosphorylation of ERK1/2 was stimulated by treatment with 10^−9^ mol/L nesfatin-1 for 2, 5, 10, 20, 30, and 60 min, with levels of phosphorylation peaking at 30 min. Pretreatment of cells with the ERK1/2-specific inhibitor PD98059 (10 μmol/L) for 30 min completely blocked nesfatin-1-induced phosphorylation of ERK1/2 (Fig. 6C and D, also Supplementary Figure S6). As shown in Fig. 6E,F,G and H, PD98059 also blocked the nesfatin-1-induced restoration of ΔΨm and suppression of caspase-3 activation. To further explore whether the C-Raf-ERK1/2 pathway was involved in the anti-apoptotic action of nesfatin-1, we assessed the effects of the C-Raf inhibitor GW5074 on ΔΨm and caspase-3 activation. After pretreatment with GW5074 (5 μmol/L) for 30 min, cells were pre-incubated with nesfatin-1 (10^−9^ mol/L) for 20 min and then incubated with MPP^+^ (300 μmol/L) for an additional 24 hrs. As shown in Fig. 6I,J,K and L, GW5074 blocked the nesfatin-1-inuduced restoration of ΔΨm and suppression of caspase-3 activation. These data suggested that nesfatin-1 antagonized MPP^+^-induced neurotoxicity by activating the C-Raf-ERK1/2 signaling pathway.

### The PKA pathway was not involved in the anti-apoptotic action of nesfatin-1

**Figure 7:**
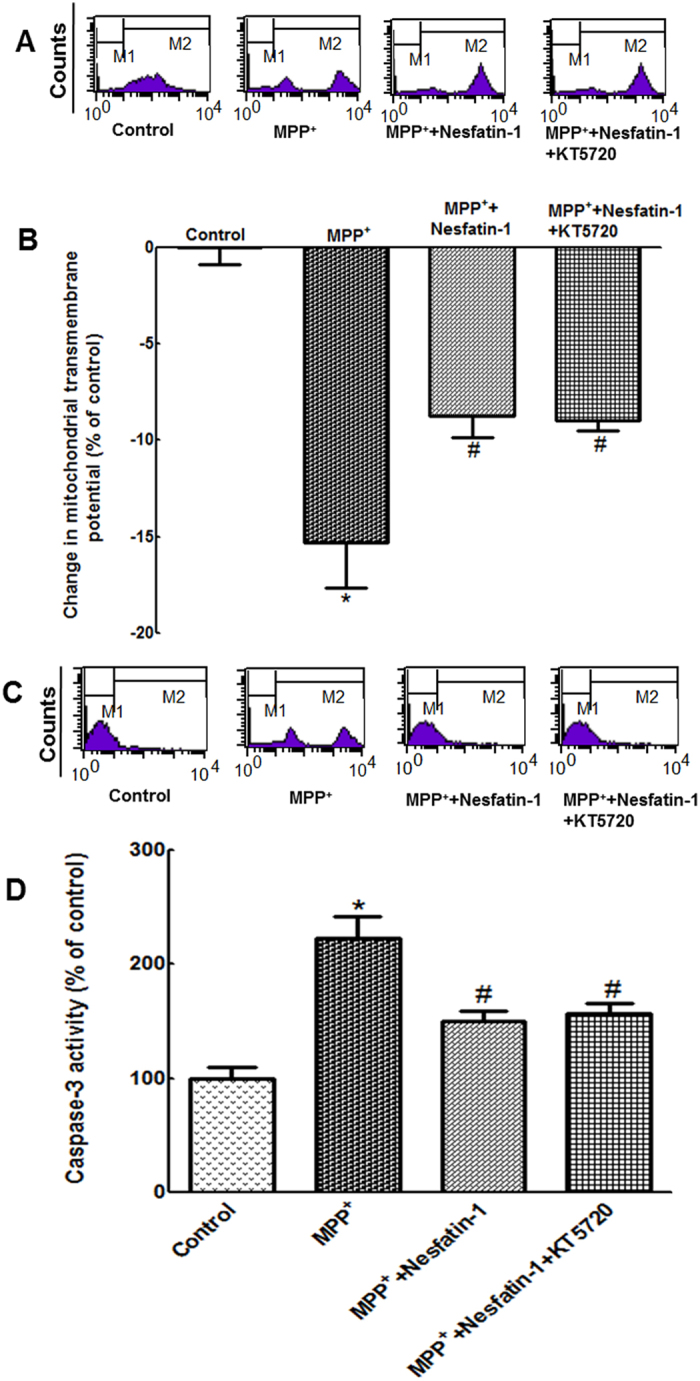
The PKA pathway was not involved in the anti-apoptotic action of nesfatin-1 in MES23.5 cells. (**A**,**B**) Pretreatment with KT5720 (1 μmol/L) did not block the protective effect of nesfatin-1 (10^−9^ mol/L) on the collapse of the ΔΨm induced by MPP^**+**^. (**C**,**D**) Pretreatment with KT5720 (1 μmol/L) did not abolish the protective effect of nesfatin-1 (10^−9^ mol/L) on caspase-3 activation induced by MPP^**+**^. ^*^P < 0.05 compared with the control group. ^#^*P* < 0.05 compared with the MPP^**+**^-treated group. (MPP^**+**^:1-methyl-4-phenylpyridillium ion, PKA: protein kinase A).

It has been reported that the effect of nesfatin-1 is mediated by increasing the intracellular Ca^2+^ levels and activating protein kinase A (PKA)^17^. To observe whether the PKA pathway participates in the anti-apoptotic action of nesfatin-1, we investigated the effect of pretreatment with the PKA inhibitor KT5720 (1 μmol/L) for 30 min on the nesfatin-1-induced restoration of ΔΨm and suppression of caspase-3 activation. As shown in Fig. 7A,B,C and D, the effects of nesfatin-1 on ΔΨm collapse and caspase-3 activation were not abolished by KT5720 pretreatment. This indicated that the PKA pathway might not be involved in the anti-apoptotic action of nesfatin-1.

## Discussion

In the present study, we demonstrated that nesfatin-1 protects dopaminergic neurons against MPTP-induced neurotoxicity in C57BL/6 mice and MPP^+^-induced cytotoxicity in MES23.5 cells, and further elucidated that the neuroprotective effect of nesfatin-1 was mediated via activation of the C-Raf-ERK1/2 signaling cascade, which inhibited apoptosis induced by mitochondrial dysfunction.

Nesfatin-1 is a satiety neuropeptide that was discovered in 2006 by Oh *et al*.^13^. It is expressed not only in neurons, including those in the forebrain, hindbrain, brainstem, and spinal cord, but also in peripheral tissues such as the gastric glands, duodenum, beta cells of the pancreatic islets, and subcutaneous fat tissue cells^12^,^19^,^35^,^36^,^37^. This novel brain-gut peptide plays an important role in hypothalamic pathways that regulate food intake and energy homeostasis^19^,^38^. Nesfatin-1 has been shown to penetrate the blood-brain barrier by a non-saturable mechanism, indicating that it is feasible for nesfatin-1 to be delivered pharmacologically to the central nervous system via a peripheral route^16^. It has been reported that nesfatin-1 has protective actions in the contexts of brain trauma and subarachnoid hemorrhage-induced injury by reducing the levels of oxidative brain injury markers and pro-apoptotic protein caspase-3 activation^26^,^27^. However, it has also been reported that 24 hours of nesfatin-1 treatment induces cardiomyocyte cell apoptosis^39^. A previous study in our laboratory demonstrated that nesfatin-1 could rescue MES23.5 cells from rotenone-induced mitochondrial dysfunction and apoptosis^30^. In the present study, we demonstrated that intracerebroventricular injection of nesfatin-1 (100, 200, 400 ng/mouse) once a day for 6 consecutive days dramatically reduced dopaminergic neuronal death and effectively prevented DA depletion in the striatum in mice treated with MPTP. We further observed that nesfatin-1 pre-incubation antagonized the MPP^+^-induced reduction in MES23.5 cell viability. Among the numerous animal models of PD, the MPTP-induced model is commonly used, as it produces clinical, biochemical, and pathological features similar to those observed in idiopathic PD^40^,^41^. MPP^+^ is the active form of MPTP and induces apoptosis by selectively damaging the mitochondrion^42^. The results of our study suggest that nesfatin-1 protects dopaminergic neurons against MPP^+^- and MPTP-induced neurodegeneration both *in vivo* and *in vitro*.

Cell apoptosis has been reported to be involved in the nigral dopaminergic neuron degeneration both in studies of post-mortem tissue from patients with PD and tissue from the MPTP-induced mouse model of PD^7^,^43^,^44^. In this process, mitochondrial dysfunction induced by complex I collapse was thought to be the primary effector^45^,^46^. MPTP inhibits complex I of the mitochondrial electron transport chain via its active form MPP^+^ and in turn induces cell apoptosis in models of PD^47^,^48^. Oxidative stress plays an important role in cell apoptosis in PD^49^,^50^. The DA-expressing areas of the brain are vulnerable to oxidative stress because DA metabolism generates reactive oxygen species (ROS)^51^,^52^. The generation and accumulation of ROS in the mitochondria leads to the opening of mitochondrial permeability transition pores and mitochondrial membrane hyperpolarization. The damaged mitochondrial membrane then leaks the pro-apoptotic protein cytochrome C into the cytoplasm. Mitochondrial cytochrome C leakage could lead to the activation of caspase-3 and nuclear fragmentation, as well as caspase-3-dependent apoptosis. Our results showed that pre-incubating MES23.5 cells with nesfatin-1 could antagonize MPP^+^-induced ΔΨm collapse, cytochrome C release from the mitochondrion into the cytoplasm, caspase-3 activation, and morphological changes in the nuclei, all of which indicated that nesfatin-1 antagonized MPP^+^ - and MPTP-induced dopaminergic neuron apoptosis by ameliorating mitochondrial dysfunction.

Although there was a report suggesting that nesfatin-1 interacted with a G-protein coupled receptor, its actual receptor has not yet been identified. Our previous study found that nesfatin-1 postsynaptically inhibited the electrical activity of nigral dopaminergic neurons, which indicated that the nesfatin-1 receptor was expressed by dopaminergic neurons^32^. The possible receptors of nesfatin-1 include melanocortin 4 receptor (MC4R), corticotropin-releasing factor type 2 receptor, and natriuretic peptide receptor A^53^,^54^,^55^. We hypothesize that the effect of nesfatin-1 on dopaminergic neurons was mediated by MC4R; further studies are required using MC4R gene-knockdown strategies to validate this hypothesis.

To elucidate the nesfatin-1-induced signaling cascades in dopaminergic neurons, specific inhibitors of PKA and ERK1/2 were used. The inhibitor of PKA did not block the protective action of nesfatin-1 on mitochondrial function, but the inhibitor of ERK1/2 abolished these protective effects. The Raf-ERK pathway is involved in a number of basic cellular processes, including cell proliferation, differentiation, and survival^56^. Our additional experiments showed that the effects of nesfatin-1 against MPP^+^-induced mitochondrial dysfunction and caspase-3 activation were abolished by pretreatment with a C-Raf inhibitor. This indicated that the activation of C-Raf-ERK1/2 pathway was involved in the protective effects of nesfatin-1 against MPP^+^-induced apoptosis in MES23.5 cells. Several nesfatin-1-induced signaling pathways have been reported. It has been documented that nesfatin-1 can potentiate glucose-induced insulin secretion by promoting Ca^2+^ influx through L-type Ca^2+^ channels in a manner independent of PKA in mouse islet β-cells^57^. It has also been reported that nesfatin-1 can stimulate the phosphorylation of AMP-dependent protein kinase (AMPK), Akt kinase (Akt) and target of rapamycin complex (TORC) 2, resulting in an increase in Fos immunoreactivity in the hypothalamic nuclei that mediate glucose homeostasis^58^. In accordance with our results, nesfatin-1 has also been reported to stimulate sympathetic nerve activity via hypothalamic ERK signaling^59^ and increased cAMP response element (CRE) reporter activity in a mouse neuroblastoma cells^60^. Further studies are required to determine which CRE-regulated genes are involved in the protective effects of nesfatin-1 on dopaminergic neurons.

**Figure 8:**
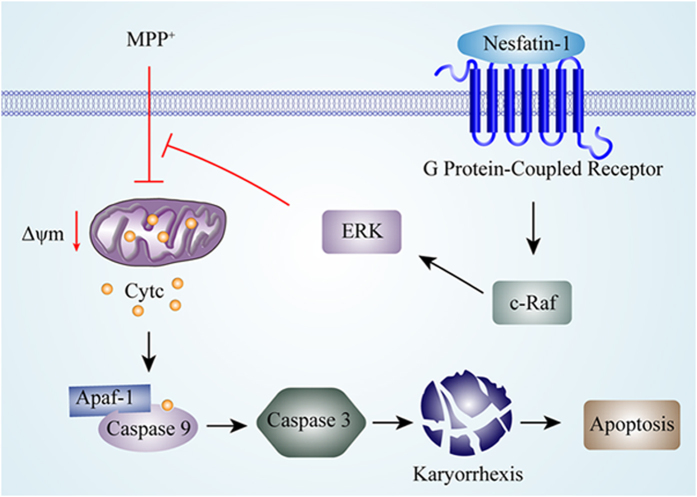
A schematic diagram of the mechanism underlying the neuroprotective effects of nesfatin-1 on dopaminergic neurons. By binding to its G-protein coupled receptor and activating the C-Raf-ERK1/2 signaling cascade, nesfatin-1 antagonizes MPP^**+**^-induced apoptosis induced by mitochondrial dysfunction in dopaminergic neurons by preventing collapse of the ΔΨm, inhibiting cytochrome C releasing from the mitochondria into the cytoplasm, interacting with Apaf-1 and caspase-9, abolishing caspase-3 activation, and attenuating karyorrhexis. (Apaf-1: apoptosis protease activating factor, ERK1/2: extracellular signal-regulated protein kinase 1/2, MPP^**+**^:1-methyl-4-phenylpyridillium ion, ΔΨm: mitochondrial transmembrane potential).

As summarized in Fig. 8, we have demonstrated the neuroprotective effect of nesfatin-1 on the MPTP-injured substantia nigra-striatum system *in vivo* and on MPP^+^-induced cytotoxicity *in vitro*. We found that this neuroprotective effect of nesfatin-1 is mediated by an anti-apoptotic C-Raf-ERK1/2 pathway that ameliorates mitochondrial dysfunction. Future basic and clinical studies of nesfatin-1 in PD are expected, and nesfatin-1 might have therapeutic potential for PD.

## Materials and Methods

### Materials

Unless otherwise stated, all chemicals were purchased from Sigma Chemical Co. (St. Louis. MO, USA). MES23.5 cells were provided by Professor Wei-Dong Le (Baylor College of Medicine, TX, USA). Nesfatin-1 was obtained from Phoenix Pharmaceuticals Inc. (Burlingame, CA, USA). Dulbecco’s modified Eagle’s medium nutrient mixture-F12 (DMEM/F12) was purchased from Gibco (Grand Island, NY, USA). The PE-conjugated monoclonal active-caspase-3 antibody apoptosis kit was from BD Bioscience (San Diego, CA, USA). PD98059 and the BCA kit was from Beyotime (Shanghai, CN). KT5720 and anti-rabbit secondary antibody conjugated to horseradish peroxidase were from Santa Cruz (Santa Cruz, CA). The cell fractionation kit was from Clontech (Mountain View, CA, USA). Anti-Cox IV monoclonal antibody was from Clontech (CA, USA). The primary antibody against TH was from Millipore (Darmstadt, Germany). Primary antibodies against ERK1/2 and phospho-ERK1/2 were from cell signaling technology (MA, USA). Alexa Fluor 555 donkey anti-rabbit secondary antibody was from Eugene (OR, USA). GW5074 was from Selleck (TX, USA).

### Animals and treatment

All procedures were performed in accordance with the National Institutes of Health Guide for the Care and Use of Laboratory Animals, and were approved by the Animal Ethics Committee of Qingdao University (20140522). Thirty healthy male C57BL/6 mice aged 10 weeks were maintained on a 12-hrs light–dark cycle at room temperature with food and water *ad libitum*. Mice were randomly divided into three groups: (1) Control group: mice received ICV saline injection only; (2) MPTP-treated group: mice received MPTP (30 mg/kg, intraperitoneal injection, i.p.) and ICV saline injections once per day for 5 consecutive days; (3) Nesfatin-1-pretreated group: mice were pretreated with different doses of nesfatin-1 (100, 200, and 400 ng/mouse, ICV) once per day for 6 consecutive days and received MPTP (30 mg/kg, i.p.) for the final 5 days. Twenty-four hours after the last injection of MPTP, all mice were sacrificed for further investigation. Brains were obtained from six mice of each group. One side of the substantia nigra was isolated to assess TH protein levels by western blotting, striatum was isolated for high-performance liquid chromatography (HPLC), and the other side of the brain was fixed in 4% paraformaldehyde (PFA) for immunofluorescent staining.

### Measurement of dopamine and its metabolites levels by HPLC

Six mice from each group provided samples for HPLC. Samples were weighed and then homogenized in 0.3 ml liquid A (0.4 M perchloric acid). After initial centrifugation (120,000 rpm for 20 min at 4 °C), 80 μl of the supernatant was transferred into Eppendorf tubes, and 40 μl liquid B (20 mM citromalic acid potassium, 300 mM dipotassium phosphate, 2 mM ethylene diaminetetraacetic acid (EDTA).2Na were added. After additional centrifugation (120,000 rpm for 20 min at 4 °C), 100 μl of the supernatant was assayed for DA and its metabolites, DOPAC and HVA, by HPLC. Separation was achieved on a PE C18 reverse-phase column. The mobile phase consisted of 20 mM citromalic acid, 50 mM sodium caproate, 0.134 mM EDTA.2Na, 3.75 mM sodium octane sulphonic acid, and 1 mM di-sec-butylamine at 5% (v/v) methanol; the flow-rate was 1 ml/min. A 2465 electrochemical detector (Waters, USA) was operated in screen mode. The results were expressed as ng/mg wet weight of brain tissue.

### Immunofluorescence and immunohistochemistry staining

Brains were fixed in 4% PFA for 72 hrs at 4 °C, followed by incubation in 0.1 M phosphate buffer (pH 7.4) containing 25% sucrose at 4 °C for 2–3 days. The frozen brain tissues were cut into 25-μm-thick sections. Brain tissue sections were used for immunofluorescence staining and stereology of the SN, and for immunohistochemical staining of the Str. The sections were first incubated with 0.1% Triton X-100 in phosphate-buffered saline (PBS) for 24 hrs and then incubated overnight at 4 °C with the TH primary antibody (1:2000) in PBS containing 0.1% Triton X-100. Sections used for staining of the SN were incubated with Alexa Fluor 555 donkey anti-rabbit as the secondary antibody, and images were obtained by immunofluorescence microscopy (Observer A1, Zeiss, Germany) with magnification at 200 and 400X. Sections used for staining of the Str were incubated with biotinylated goat anti-rabbit IgG for 1 hrs at 37 °C, followed by amplification with streptavidin peroxidase for 1 hrs at 37 °C. Diaminobenzidine hydrogen peroxide (0.01%) was used as the chromogen, and digital images were obtained using a camera.

### Stereological analysis

The total number of TH +  neurons was estimated bilaterally every 4th section through the extent of the substantia nigra of each brain. Stereology was performed at 400X using a Axioplan 2 Imaging microscope (Zeiss, Göttingen, Germany), fitted with a DEI-750 CE video camera (Optronics, California, USA) and a LEP MAC5000 motorized stage controller (Ludl Electronic Products, New York, USA). The software package was Stereo Investigator (MBF Bioscience, Vermont, USA). The coefficient of error for the individual counts was 0.01. Data were expressed as TH + neurons/substantia nigra.

### Measurement of MPP^+^ levels by HPLC

Samples of the striatum were homogenized in 0.1 ml phosphate-buffered saline, and 0.1 ml of cold acetonitrile was added to precipitate proteins, then the mixtures were centrifuged in 10000 rpm for 5 min. To determine the concentration of MPP^**+**^, the supernatant fractions were analyzed by LC-6A HPLC system (Shimadzu, Japan) and hypersil ODS_2_ C18 column(Shimadzu, Japan). The mobile phase was 85% (v/v) acetonitril in 0.1 mol/L acetic acid (pH = 5.6). The flow rate was 1.3 ml/min. The detection wave length was 293 nm.

### Cell culture and treatments

As previously described by our laboratory, MES23.5 cells were cultured in DMEM/F12 with 5% heat-inactivated fetal bovine serum and Sato components, and maintained at 37 °C under a humidified atmosphere of 95% air and 5% CO_2_^9^,^61^. For the experiments, cells were seeded at a density of 1 × 10^5^/cm^2^ in plastic flasks or plates. To detect cell viability, cells were pre-incubated with 10^−7^ to 10^−14^ mol/L nesfatin-1 for 20 min and then incubated with MPP^**+**^ (300 μmol/L) for another 24 hrs. To determine the extent of mitochondrial protection and the anti-apoptotic effects of nesfatin-1, cells were pretreated with nesfatin-1 (10^−9^ mol/L) for 20 min and then incubated with MPP^**+**^ (300 μmol/L) for 24 hrs. To investigate the involvement of the C-Raf-ERK1/2 pathway in the anti-apoptotic effects of nesfatin-1, cells were pretreated with PD98059 (10 μmol/L) or GW5074 (5 μmol/L) for 20 min prior to the addition of nesfatin-1 (10^−9^ mol/L). After 20 min, cells were incubated with MPP^**+**^ for 24 hrs.

### Methyl thiazolyltetrazolium (MTT) assay and Hoechst 33258 staining

To assess the neuroprotective effects of nesfatin-1 against MPP^**+**^ -induced cytotoxicity, MES23.5 cells were treated as outlined above. After 24 hrs incubation, cells were incubated with methyl thiazolyltetrazolium (MTT; 5 mg/mL) for a further 4 hrs at 37 °C. Cell viability was assessed at 494 nm and 630 nm with a spectrophotometer (Tecan, Grodig, Austria). We then added nesfatin-1 (10^−9^ mol/L) and observed nuclear morphology using a previously described method^62^. Briefly, cells were fixed in 4% PFA for 30 min, washed in PBS, and stained with Hoechst 33258 dye for 15 min at room temperature. After washing three times to remove the excess dye, cells were examined and photographed using a confocal laser scanning microscope (Fluoview FV500, Olympus, Japan).

Apoptotic cells were defined on the basis of nuclear morphological changes, such as chromatin condensation and fragmentation. For each well, all condensed and normal nuclei were counted in at least 10 different fields. The average sum of condensed and normal nuclei was calculated per well. The proportion of nuclei that were condensed relative to the total number of nuclei was calculated, and the data are presented as percentages.

### Assessment of ΔΨm and caspase-3 activation

To determine ΔΨm, cells were incubated with rhodamine-123 (Sigma, Missouri, USA, 5 μmol/L) for 15 min. After washing twice with 2-[4-(2-Hydroxyethyl)-1-piperazinyl] ethanesulfonic acid (HEPES)-buffered saline, the fluorescence intensity was recorded at 488 nm excitation and 525 nm emission by flow cytometry. Caspase-3 activity was measured by flow cytometry using a PE-conjugated monoclonal active caspase-3 antibody apoptosis kit. Cells were resuspended in 500 μL Citofix/Cytoperm™ and incubated on ice for 20 min. Cells were then washed twice with Perm/Wash buffer and incubated with antibody (100 μL Perm/Wash buffer plus 10 μL antibody per sample) for 30 min. After one wash with Perm/Wash buffer, cells were resuspended in 0.5 mL Perm/Wash buffer and analyzed by flow cytometry at 523 nm excitation and 658 nm emissions. Apoptosis was evaluated as the percentage of caspase-3-immunoreactive cells out of the total number of cells using Cellquest Software (BD Bioscience).

### Western blotting

Samples from cells and animals were lysed in a buffer containing 50 mmol/L Tris-HCl, 150 mmol/L NaCl, 1% Nonidet P-40, 0.5% sodium deoxycholate, 1 mmol/L EDTA, and a protease inhibitor cocktail. The protein concentration was determined using BCA kits. Eighty milligram of total protein were separated by 10% SDS–polyacrylamide electrophoresis (SDS-PAGE) gels and then transferred onto PVDF membranes. After blocking with 10% non-fat milk at room temperature for 2 hrs, the membranes were incubated overnight at 4 °C with antibody against ERK1/2 (rabbit, 1:1000), phospho-ERK1/2 (rabbit, 1:1000), or TH (rabbit, 1:4000). The membranes were then incubated with anti-rabbit secondary antibody conjugated to horseradish peroxidase (1:10,000). Cross-reactivity was visualized using ECL western blotting detection reagents and then analyzed via scanning densitometry using a Tanon image system. β-actin was detected via rabbit anti-β-actin monoclonal antibody (1:10,000) using the same procedure.

### Mitochondria isolation and detection of cytochrome C release from the mitochondria into cytoplasm

After pretreatment with nesfatin-1 (10^−9^ mol/L) for 20 min, cells were incubated with MPP^**+**^ (300 μmol/L) for 24 hrs. The extraction and isolation of mitochondrial and cytoplasmic proteins was performed using the cell fractionation kit according to the manufacturer’s instructions. Samples were separated on a 12% SDS-PAGE gel. Protocols for using the anti-cytochrome C monoclonal antibody and anti-Cox IV monoclonal antibody were provided as appendices of the manufacturer’s instructions in the cell fractionation kit. Blots were probed with anti-β-actin monoclonal antibody (1:5,000) as a loading control for cytoplasmic samples and with anti-Cox IV monoclonal antibody (1:800) as a loading control for mitochondrial samples.

### Statistical analysis

SPSS 18.0 (SPSS Inc, Chicago, IL, USA) was used to analyze the data. All data are shown as mean ± SEM. Differences between the means of two groups were compared using the unpaired-samples T test. One-way analysis of variance (ANOVA) followed by the Student-Newman-Keuls test was used to compare differences between means in more than two groups. *P* < 0.05 was considered statistically significant.

## Additional Information

**How to cite this article**: Shen, X.-L. *et al*. Nesfatin-1 protects dopaminergic neurons against MPP^+^/MPTP-induced neurotoxicity through the C-Raf–ERK1/2-dependent anti-apoptotic pathway. *Sci. Rep.* **7**, 40961; doi: 10.1038/srep40961 (2017).

**Publisher's note:** Springer Nature remains neutral with regard to jurisdictional claims in published maps and institutional affiliations.

## Supplementary Material

Supplement Figure
