# Supplementary material for: Nesfatin-1 protects dopaminergic neurons against MPP+/MPTP-induced neurotoxicity through the C-Raf–ERK1/2-dependent anti-apoptotic pathway
Source: Sci Rep. 2017 Jan 20;7:40961. doi: 10.1038/srep40961 (PMC5247731; doi:10.1038/srep40961)
Supplement: Supplement Figure [file srep40961-s1.pdf]

Nesfatin-1 protects dopaminergic neurons against MPP^+/MPTP-induced neurotoxicity through the C-Raf–ERK1/2-dependent anti-apoptotic pathway

Xiao-Li SHEN[1], Ning SONG[1], Xi-Xun DU[1], Yong LI[1], Jun-Xia XIE[1], *Hong JIANG[1]

1 Department of Physiology, Shandong Provincial Key Laboratory of Pathogenesis and Prevention of Neurological Disorders and State Key Disciplines: Physiology, Medical College of Qingdao University, Qingdao, 266071, China

* Address correspondence to:

Hong Jiang (professor)

Department of Physiology, Medical College of Qingdao University, Qingdao, 266071, China.

Tel: 86-532-83780191

Fax: 86-532-83780136

EMAIL: hongjiang@qdu.edu.cn (H. Jiang)

SUPPLEMENTARY INFORMATION includes:

Supplementary Figures S2，S5，S6

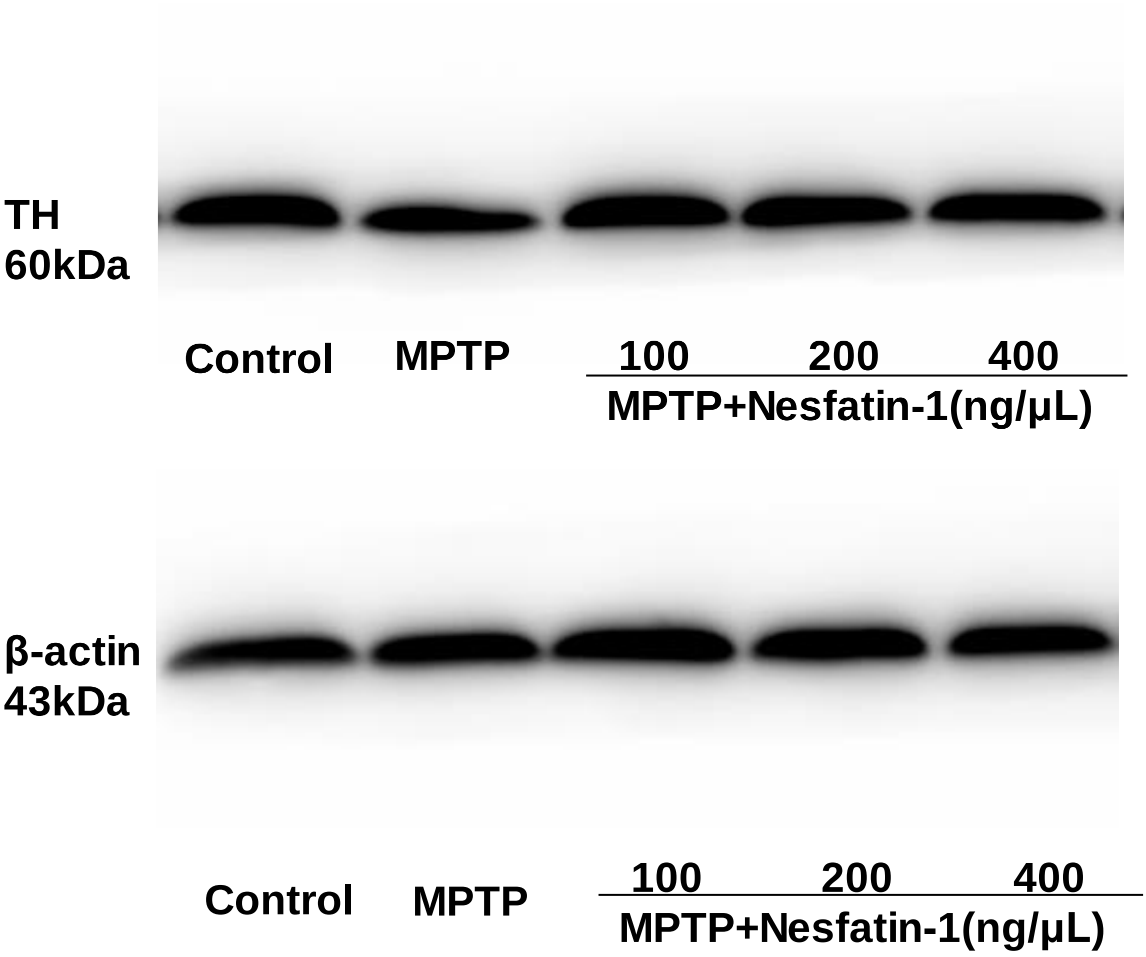

Supplementary Figure S2: Western blots showing the different TH and β-actin levels in control, MPTP and MPTP+Nesfatin-1 group.

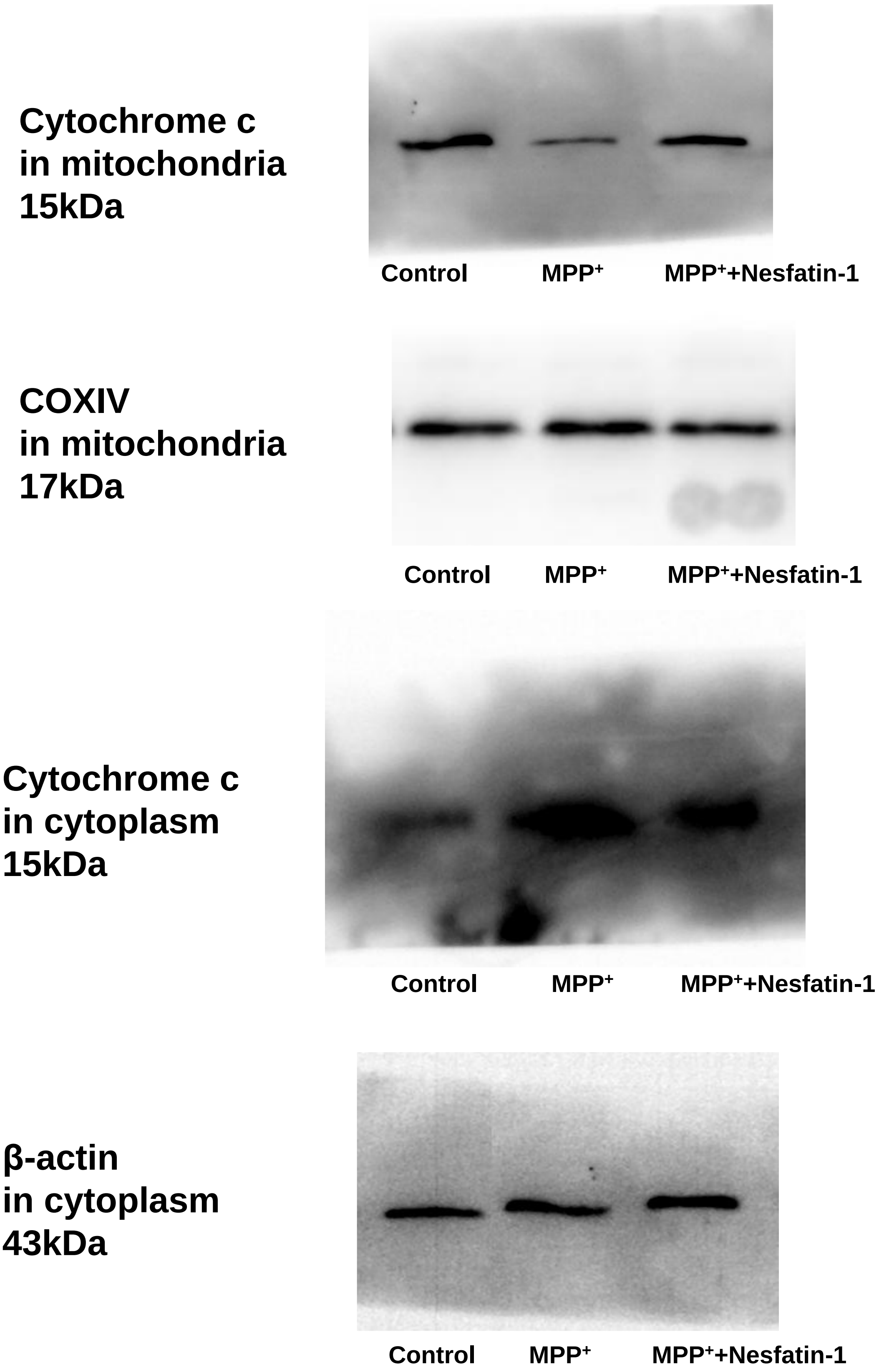

Supplementary Figure S5: Western blots showing the Cytochrome c, COXIV, β-actin levels in control, MPP^+ and MPP^++Nesfatin-1 group in the mitochondria and cytoplasm.

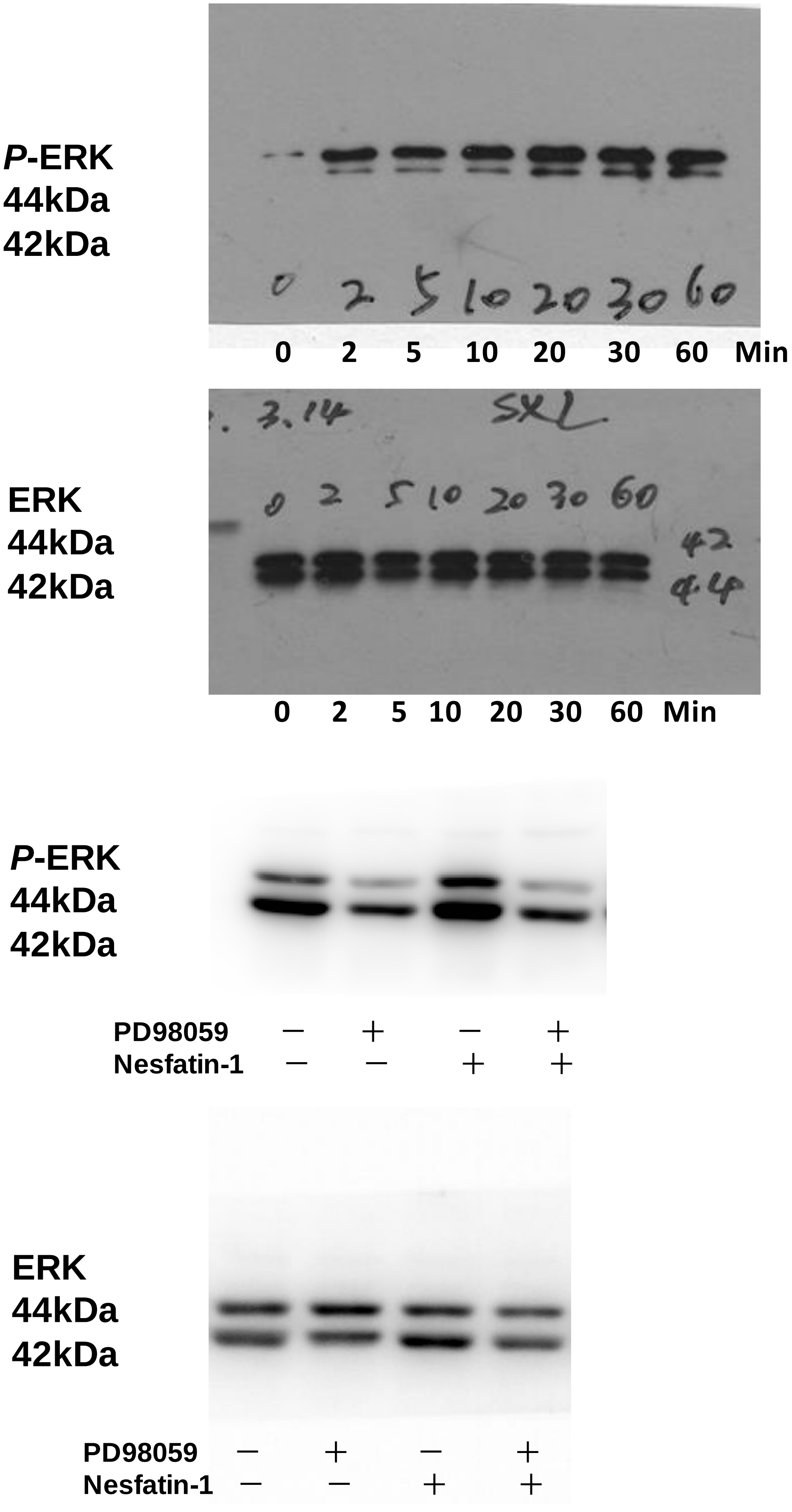

Supplementary Figure S6: Western blots showing the ERK, *p*-ERK, levels changes after treatment with nesfatin-1 and PD98059.
